# Metasurface for complete measurement of polarization Bell state

**DOI:** 10.1515/nanoph-2022-0593

**Published:** 2022-11-21

**Authors:** Zhanjie Gao, Zengping Su, Qinghua Song, Patrice Genevet, Konstantin E. Dorfman

**Affiliations:** State Key Laboratory of Precision Spectroscopy, East China Normal University, Shanghai 200062, China; Tsinghua Shenzhen International Graduate School, Tsinghua University, Shenzhen 518055, China; Université Côte d’Azur, CNRS, CRHEA, Rue Bernard Gregory, Valbonne 06560, France; Collaborative Innovation Center of Extreme Optics, Shanxi University, Taiyuan, Shanxi 030006, China; Himalayan Institute for Advanced Study, Unit of Gopinath Seva Foundation, MIG 38, Avas Vikas, Rishikesh, Uttarakhand 249201, India

**Keywords:** Bell state, metasurface, quantum entanglement

## Abstract

Bell state measurement is vital to quantum information technology. Conventional linear optical elements, however, cannot fully distinguish all polarization Bell states without assisting of additional degrees of freedom. Leveraging on a pair of binary-pixel metasurfaces, we demonstrate direct measurement of all four polarization Bell states. Each metasurface is designed to produce two output modes that linearly superpose three Bell states in the coincidence counting measurement. By rotating the polarizers, the coincidence counting measurement achieves a tunable anticorrelation between one and the other two Bell states, achieving Bell state detection efficiency of 75% in a single measurement. Complete and deterministic Bell state measurement is further realized by performing two measurements. Our work shows the advantage of utilization of metasurfaces in quantum detection schemes and is of great applicative interest for quantum dense coding, entanglement swapping, quantum teleportation protocols, and novel quantum information processing tasks.

## 1 Introduction

Bell states measurement (BSM) is essential for a broad range of quantum information applications, including quantum dense coding [1, 2], entanglement swapping [3, 4], quantum teleportation protocols [5, 6], quantum secure direct communication [7, 8], etc. However, according to the no-go theorem for the Bell analyzer, it is not possible to distinguish all four Bell states using only conventional linear optical elements or without invoking additional degrees of freedom [9, 10]. For example, BSM efficiency of the most common Bell states analyzer based on the Innsbruck scheme consisting of one 50:50 beam splitter and two polarizing beam splitters (PBS) is no more than 50% [11], [12], [13], [14]. With the help of auxiliary photons, the discrimination probability of the Innsbruck scheme can be improved to 75%, but a complete BSM has never been reported [15, 16]. To achieve perfect BSM, hyperentanglement embedding Bell states in a larger Hilbert space is widely applied to distinguish all Bell states [17], [18], [19], [20]. Hyperentanglement schemes require additional degrees of freedom and extra quantum resources. The complete measurement can also be achieved using relatively inefficient nonlinear process, which destroys the original state of the photon [21], [22], [23]. A new complete BSM scheme that would go beyond the hyperentanglement and nonlinear regime is thus still needed.

Here, we show that the metasurfaces, specifically designed to simultaneously transmit different polarization in two output channels, provide unprecedented opportunities to solve this problem. Metasurfaces, as a two-dimensional artificial metamaterial, provides a flexible designable optical platform to arbitrarily control the optical wavefront, orbital angular momentum, and polarization of the light [24], [25], [26], [27], [28], [29]. Recently, metasurfaces operating in quantum regime have been demonstrated [30, 31], with applications including generation of nonclassical light [32], [33], [34], [35], [36], control of quantum interference [37, 38], quantum-enhanced sensing and quantum imaging [39], [40], [41], etc. These developments and the design advantages are positioning the metasurface technology for applications in quantum optics and particularly for the control of entanglement states. In addition, the geometric phase metasurfaces have been utilized to generate path-entangled two-photon NOON state [42] and trigger the entanglement between the spin and the orbital angular momentum of photons [43]. Utilizing the polarization control capability of metasurfaces [44], it enabled a complete quantum state tomography [45], multichannel entanglement distribution and transformation [46]. However, as an essential building block of quantum information processing, a complete metasurface-based BSM setup without any aid of other auxiliary photons or degrees of freedom has not yet been explored.

In this work, we propose a specific design of binary-pixel metasurfaces to fully distinguish all four Bell states. In our approach, the PBS in the Innsbruck BSM scheme is replaced by metasurfaces composed of different pixels which allows to realize the polarization control in the output channels. Theoretical derivations expressing the effective Hamiltonian for the linear metasurfaces are derived, providing the relation between the input and the output channels. Using the quantum description of PB metasurfaces, we theoretically demonstrate that the metasurface-enhanced Innsbruck scheme achieves a complete BSM by adjusting the rotation angles of the polarizers before the photon detectors. Finally, we show the projection measurement for the input two-photon quantum states.

## 2 Quantum description of metasurface-based polarization beam splitter

**Figure 1:**
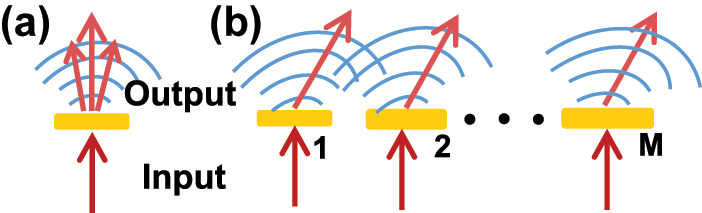
Sketch for the transformation of photon states by the single nano-antenna (a) and periodic supercell with *M* antennas (b). In (b), each antenna has a designed geometric shape providing a desired phase and amplitude configuration to control the path and the polarization degree of freedom of photon.

To describe the quantum transformation of photon states by the metasurface, we define the metasurface as a basic linear operator composed by an assembly of oriented single anisotropic antennas shown in Figure 1(a). The response of an anisotropic antennas aligned along *x* and *y* coordinates can be represented by the effective susceptibility matrix $\chi =\left(\begin{array}{cc} \chi_{xx} & 0\\ 0 & \chi_{yy} \end{array}\right)$, where *χ*_*xx*_ and *χ*_*yy*_ are the complex effective susceptibility coefficients. In the following, the quantum states of light are defined by their quantized electric fields according to

(1) $$\begin{array}{l} \begin{array}{cc} {\hat{E}}_{k,g}\left(t,r\right) & ={\hat{E}}_{k,g}^{+}\left(t,r\right)+{\hat{E}}_{k,g}^{-}\left(t,r\right)\\ & =\sqrt{\frac{\hbar \omega_{k}}{2\epsilon_{0}V}}\left({\hat{a}}_{k,g}e^{ik\cdot r-i\omega t}+{\hat{a}}_{k,g}^{†}e^{-ik\cdot r+i\omega t}\right)e_{x}, \end{array} \end{array}$$

where *ϵ*_0_ is the vacuum permittivity, *ω*_*k*_ is the frequency corresponding to ***k***, and *V* is the quantization volume. ${\hat{a}}_{k,g}$, ${\hat{a}}_{k,g}^{†}$ are the annihilation and the creation operators for the ***k*** mode photon with *g*− polarization, respectively. The effective Hamiltonian describing the transport of photons across a single antenna in a linear optical process is thus given by

(2) $$\begin{array}{c} {\hat{H}}_{\text{eff}}^{\prime }\left(t\right)=\underset{V}{\int }dr\overset{y}{\underset{f,g=x}{\sum }}\epsilon_{0}\chi_{gf}{\hat{E}}_{k^{\prime },g}^{-}\left(t,r\right)E_{k,f}^{+}\left(t,r\right)+H.c., \end{array}$$

where ***k*** and ***k*** ′ represent the wave vectors for the input and the scattering light, respectively.

Consider a metasurface composed of uniformly distributed *M* anisotropic antennas in a *Ma*_*x*_ × *a*_*y*_ supercell as shown in 1(b), then the corresponding lattice primitive translation is ***ϱ***_*m*,*n*_ = *mM* ***a***_*x*_ + *n* ***a***_*y*_ and reciprocal lattice is $G_{mn}=\frac{2\pi m}{a_{x}}e_{x}+\frac{2\pi n}{a_{y}}e_{y}$, where *a*_*x*_ = | ***a***_*x*_| and *a*_*y*_ = | ***a***_*y*_| are the antenna spacing along the *x* and the *y* direction, respectively. The path mode ***k*** and polarization *g* of the scattered photon are controlled by the spatial profile of scattered amplitude and the phase originated from the arrangement of antennas with different shapes. We start with the scattered photon path mode ***k*** and polarization *g* of photon transmitted across the metasurface with an arbitrary periodic configuration.

Assembling various anisotropic antennas of different shapes in an array enables the space-dependent effective susceptibility $X_{gf}\left(r\right)=\sum_{j,l=-\infty }^{\infty }\sum_{j=0}^{M}\chi_{gf}^{j^{\prime }}\delta \left(r-\rho_{j,j^{\prime },l}\right)$, where ***ρ***_*j*,*j*′,*l*_ = ***ϱ***_*m*,*n*_ − ***ρ***_*j*′_ denotes the *j*′ antenna in the (*j*, *l*) supercell at the inner location ***ρ***_*j*′_ = *j*′*a*_*x*_ ***e***_*x*_. In the following, each antenna is approximated as a point-shape object, expressing the effects of their detailed geometrical shapes though their effective susceptibility. In this way, the amplitude and phase factor can be expressed by the effective susceptibility $\chi_{gf}^{j^{\prime }}$ as $\chi_{gf}^{j^{\prime }}=|\chi_{gf}^{j^{\prime }}|{\text{e}}^{\text{i}\phi_{gf}^{j^{\prime }}}$. The corresponding effective Hamiltonian associated with the metasurface reads as

(3) $$\begin{array}{l} \begin{array}{cc} {\hat{H}}_{\text{eff}}\left(t\right) & =\underset{V}{\int }dr\overset{M}{\underset{j^{\prime }=0}{\sum }}\overset{\infty }{\underset{j,l=-\infty }{\sum }}\overset{y}{\underset{f,g=x}{\sum }}\delta \left(r-\rho_{j,j^{\prime },l}\right)\\ & \;\times \epsilon_{0}|\chi_{gf}^{j^{\prime }}|{\text{e}}^{\text{i}\phi_{gf}^{j^{\prime }}}{\hat{E}}_{k^{\prime },g}^{-}\left(t,r\right)E_{k,f}^{+}\left(t,r\right)+H.c. \end{array} \end{array}$$

Using the Poisson summation $\sum_{j,l=-\infty }^{\infty }\delta \left(r-\rho_{j,j^{\prime },l}\right)=\sum_{m,n=-\infty }^{\infty }{\text{e}}^{\text{i}G_{m,n}\left(r+\rho_{j^{\prime }}\right)}$ and performing the spatial integration, the effective Hamiltonian (3) can be recast as

(4) $$\begin{array}{l} \begin{array}{cc} {\hat{H}}_{\text{eff}}\left(t\right) & =\overset{\infty }{\underset{m,n=-\infty }{\sum }}\overset{y}{\underset{f,g=x}{\sum }}F_{gf}a_{k^{\prime },g}^{†}{\hat{a}}_{k,f}\\ & \;\times {\text{e}}^{-\text{i}\left(\omega -\omega^{\prime }\right)t}\delta \left(k+G_{m,n}-k^{\prime }\right)+H.c., \end{array} \end{array}$$

where the scattering factor is $F_{gf}=\sum_{j^{\prime }=0}^{M}\epsilon_{0}|\chi_{gf}^{j^{\prime }}|{\text{e}}^{\text{i}\left(G_{m,n}\rho_{j^{\prime }}+\phi_{gf}^{j^{\prime }}\right)}$. We next consider the evolution of photon states interacting and transmitting across the metasurface. In the first-order perturbation approximation, the output state of photon is

(5) $$\begin{array}{l} \begin{array}{cc} |\Psi 〉 & =-\frac{i}{\hbar }\int dt{\hat{H}}_{\text{eff}}\left(t\right)|\Psi_{in}〉\\ -\frac{i}{\hbar } & =\overset{\infty }{\underset{m,n=-\infty }{\sum }}\overset{y}{\underset{f,g=x}{\sum }}F_{gf}a_{k^{\prime },g}^{†}{\hat{a}}_{k,f}\\ & \;\times \delta \left(k+G_{m,n}-k^{\prime }\right)\delta \left(\omega -\omega^{\prime }\right)|{0〉}_{s}|{\psi 〉}_{in}, \end{array} \end{array}$$

where initial state of the scattering (output) mode is in vacuum state |0⟩_*s*_, and the initial state of the input photon is ${|\psi 〉}_{in}=\alpha {|n_{x}〉}_{in}+\beta {|n_{y}〉}_{in}$. Here, *n*_*x*_ and *n*_*y*_ correspond to the number of photon in *x* and *y* polarization, respectively. The scattering light acquires an additional momentum from the periodic structure, which results in the different output paths analogous to the Bragg scattering in solid crystals and gives rise to the generalized laws of reflection and refraction [28, 47]. As shown in Eq. (5), the control of different paths and polarization modes is determined by the scattering factor *F*_*gf*_.

**Figure 2:**
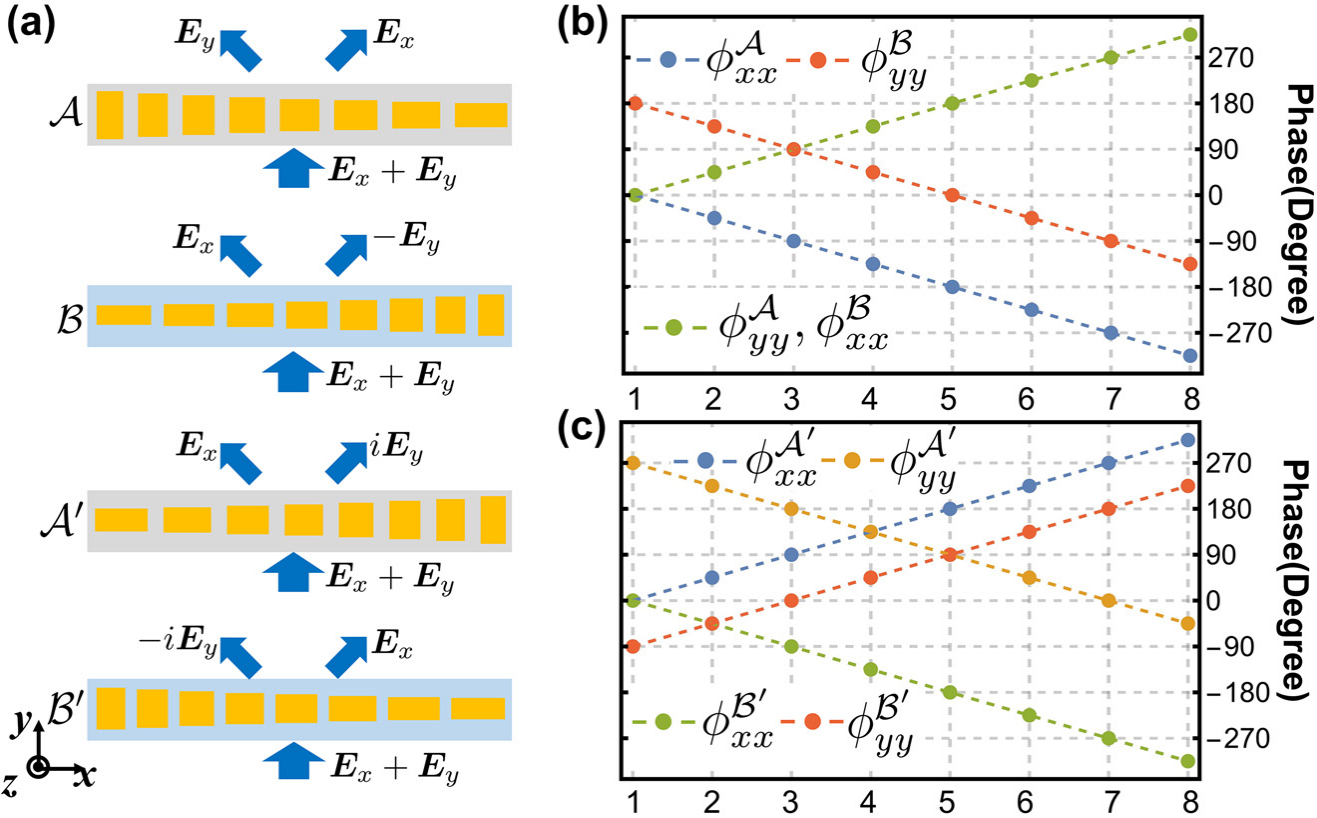
Design principle of four phase gradient supercells for binary-pixel metasurface, denoting as A, B, $A^{\prime }$, and $B^{\prime }$. (a) The combination of pixel consisting supercell A and pixel consisting supercell B can convert the incident light ***E***_*x*_ + ***E***_*y*_ to ***E***_*x*_ + ***E***_*y*_ to the right and ***E***_*x*_ − ***E***_*y*_ to the left. The combination of two pixels consisting supercell $A^{\prime }$ or $B^{\prime }$ convert the incident light ***E***_*x*_ + ***E***_*y*_ to ***E***_*x*_ + *i* ***E***_*y*_ to the right and ***E***_*x*_ − *i* ***E***_*y*_ to the left. (b) The supercell A has opposite linear phase gradient for $\phi_{xx}^{A}$ and $\phi_{yy}^{A}$. The phase gradient of $\phi_{xx}^{B}$ and $\phi_{yy}^{B}$ of supercell B is same as that of $\phi_{yy}^{A}$ and $\phi_{xx}^{A}$, while there is phase delay $\phi_{yy}^{B}-\phi_{xx}^{A}=\pi$. (c) For metasurface M2, $\phi_{xx}^{A^{\prime }}$ and $\phi_{yy}^{B^{\prime }}$ have the opposite linear gradient. The phase delay between binary-pixel consisting supercell $A^{\prime }$ and $B^{\prime }$ satisfies $\phi_{xx}^{B^{\prime }}-\phi_{yy}^{A^{\prime }}=-3\pi /2$ and $\phi_{yy}^{B^{\prime }}-\phi_{xx}^{A^{\prime }}=-\pi /2$.

In the conventional Innsbruck scheme, the coincidence counting measurements of two output modes of the beam splitter (BS) distinguishes the antisymmetric Bell state. A main limitation of conventional PBS is that it only splits the photon into horizontally and vertically polarized states. To distinguish the remaining two symmetric Bell states, the traditional method consists of a composite optical system that splits the photon into the different linear superpositions of polarization states by rotating the PBS and moving in or removing other linear optical devices such as the half-wave plates. This composite optical system includes the cascaded optical elements to perform various successive measurements, making the whole experimental setup too complex and non robust. More importantly, in these conventional setup, it is impossible to realize the complete measurement of all Bell states [9, 10]. In the following metasurface-enhanced BSM, two different binary-pixel metasurfaces are used to realize the different functions in an integrated setup, which results in the complete measurement of all Bell states. These two different binary-pixel metasurfaces shown in Figure 2 are designed to meet the demand of complete measurement of all four polarization Bell states.

The binary-pixel metasurface *M*1 (*M*2) is consisted of two pixels with supercell $A\;\left(A^{\prime }\right)$ and $B\;\left(B^{\prime }\right)$ as shown in Figure 2(a). Note that there are several rows of supercells in each pixel, which helps the metasurface to avoid the undesirable interference between the two pixels. In fact, only ±1 diffraction orders in *x* direction contribute to the light transmission. Thus, we assume *λ* < *Ma*_*x*_ < 5*λ* and *a*_*y*_ < *λ* where *λ* is the wavelength of the incident light. The anisotropic antennas in each pixel are uniformly distributed in space. In each pixel, the amplitude response of every antenna is the same $\left|\chi_{xx}^{I}\right|$ and $|\chi_{yy}^{I}|\;\left(I=A,A^{\prime },B,B^{\prime }\right)$, while their phase response $\phi_{gf}^{j^{\prime }}$ changes linearly from 0 to ±2*π* with different linear phase gradient. Hereafter, $\left|\chi_{gf}^{I}\right|$ and $\phi_{gf}^{I}$ are used to denote the same amplitude response and the entire phase distribution in each pixel, respectively.

For the metasurface *M*1 shown in Figure 2(b), the phase distributions $\phi_{xx}^{A}$ and $\phi_{yy}^{B}$ have a negative gradient with a phase delay $\phi_{yy}^{B}-\phi_{xx}^{A}=\pi$. The phase distributions $\phi_{yy}^{A}$ and $\phi_{xx}^{B}$ are opposite to $\phi_{xx}^{A}$ and have no phase delay compared to $\phi_{xx}^{A}$. These uniformly phase distributions result in the scattering factor *F*_*gf*_ to vanish for the higher scattering orders {*m* ≠ ±1, *n* ≠ 0}. Thus, the state of photon scattered by the metasurface *M*1 can be recast as Eq. (6).

(6) $$\begin{array}{l} \begin{array}{cc} |\Psi 〉= & -\frac{i5\epsilon_{0}}{\hbar }\delta \left(\omega -\omega^{\prime }\right)\left\{\left[|\chi_{xx}^{A}|a_{k^{\prime },x}^{†}{\hat{a}}_{k,x}-|\chi_{yy}^{B}|a_{k^{\prime },y}^{†}{\hat{a}}_{k,y}\right]\right.\\ & \times \;\delta \left(k+G_{1,0}-k^{\prime }\right)+\left[|\chi_{xx}^{B}|a_{k^{\prime },x}^{†}{\hat{a}}_{k,x}+|\chi_{yy}^{A}|a_{k^{\prime },y}^{†}{\hat{a}}_{k,y}\right]\\ & \times \;\left.\delta \left(k+G_{-1,0}-k^{\prime }\right)\right\}{|0〉}_{s}{|\psi 〉}_{in}, \end{array} \end{array}$$

Equation (6) reveals that there are two output paths with *m* = ±1. The first line of Eq. (6) shows that the *x* − (*y*−) polarized photon is created in *m* = 1 path when *x* − (*y*−) polarized incident photon is annihilated. There is an extra *π* phase delay between the *m* = 1 path and the incident path for the *y*− polarized photon. Meanwhile, the detection probability of *x*− and *y*− polarized photon in each path is proportional to the amplitude response coefficient $|\chi_{gf}^{I}{|}^{2}$ with I=A,B. To improve the work efficiency in the BSM scheme in the following part, we assume that the designed metasurfaces have an ideal transmission without absorption and reflection. Considering the photon number conservation and normalization, the relation between the photons in the input and the ±1st order channels can be written as

(7) $$\begin{array}{l} \begin{array}{cccc} {\hat{a}}_{1,x} & =\cos\alpha_{1}{\hat{a}}_{x}, & \;{\hat{a}}_{1,y} & =-\cos\alpha_{2}{\hat{a}}_{y},\\ {\hat{a}}_{-1,x} & =\sin\alpha_{1}{\hat{a}}_{x}, & \;{\hat{a}}_{-1,y} & =\sin\alpha_{2}{\hat{a}}_{y}, \end{array} \end{array}$$

where the angle *α*_*i*_ (*i* = 1, 2) in the amplitude coefficients varies from 0 to *π*/2. Here, we denote all the annihilation operators ${\hat{a}}_{x,y}$ as the annihilation of *x*−, *y*− polarized photons, and index ±1 corresponds the different scattering channels with wave vectors ***k*** ′ = ***k*** + ***G***_±1,0_.

For the metasurface *M*2, the phase distribution in different pixel satisfies $\phi_{yy}^{B^{\prime }}-\phi_{xx}^{A^{\prime }}=-\pi /2$ and $\phi_{xx}^{B^{\prime }}-\phi_{yy}^{A^{\prime }}=-3\pi /2$ as shown in Figure 2(c). Such phase distribution leads to an additional *π*/2(3*π*/2) phase delay between the created *m* = 1(−1) path photon and the annihilated incident path for the *y*− polarized photon. Thus, the input–output relation for the *M*2 metasurface reads

(8) $$\begin{array}{l} \begin{array}{cccc} {\hat{a}}_{1,x} & =\cos\alpha_{3}{\hat{a}}_{x}, & \;{\hat{a}}_{1,y} & =-i\cos\alpha_{4}{\hat{a}}_{y},\\ {\hat{a}}_{-1,x} & =\sin\alpha_{3}{\hat{a}}_{x}, & \;{\hat{a}}_{-1,y} & =i\sin\alpha_{4}{\hat{a}}_{y}. \end{array} \end{array}$$

**Figure 3:**
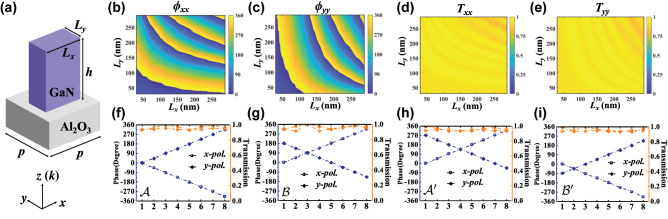
Gallium nitride (GaN) nanopillars with simulated phase and transmission at the wavelength of 700 nm with normal incidence. (a) Schematic illustration of a GaN nanopillar on a sapphire substrate. (c–e) The simulation results for the phase (*ϕ*_*xx*_ and *ϕ*_*yy*_) and amplitude (*T*_*xx*_ and *T*_*yy*_) of the transmission coefficients. The width (*L*_*x*_) and length (*L*_*y*_) of the GaN nanopillars are both swept in the range of 0–290 nm. The selected transmittance and phase of each supercell for the design of pixels with supercells A (f), B (g), $A^{\prime }$ (h), and $B^{\prime }$ (i).

To put the above metasurface design into practical perspective, we exploit the numerical simulation using the Lumerical finite-difference time-domain (FDTD) method. As shown in Figure 3(a), the basic unit cell of the metasurface is a cuboid gallium nitride (GaN) nanopillar on top of sapphire (Al_2_O_3_) substrate. The width *p* of the basic element is 300 nm. The height *h* of GaN nanopillar is 2 μm. The width and length of GaN nanopillar are denoted as *L*_*x*_ and *L*_*y*_, respectively. The selected operating wavelength is 700 nm. To realize the desired phase gradient arrangement shown in Figure 2(b) and (c), we calculate the phase (*ϕ*_*xx*_ and *ϕ*_*yy*_) and the transmission (*T*_*xx*_ and *T*_*yy*_) of GaN nanopillar for the *x*− and *y*− polarized illumination. As shown in Figure 3(b) and (c), both phases *ϕ*_*xx*_ and *ϕ*_*yy*_ can cover the entire (0, 2*π*) range and all possible combinations of *ϕ*_*xx*_ and *ϕ*_*yy*_ can be found. Besides, a near-unity amplitude distribution with high efficiency can be achieved for all values of *L*_*x*_ and *L*_*y*_. We need to carefully select the width (*L*_*x*_) and length (*L*_*y*_) of each GaN nanopillar to realize the phase gradient as shown in Figure 2(b) and (c). According to the results of Figure 3(a)–(e), eight resonators with high transmittance and desired phase configuration can be found for the pixels with supercells A, B, $A^{\prime }$, and $B^{\prime }$, as shown in Figure 3(f)–(i).

**Figure 4:**
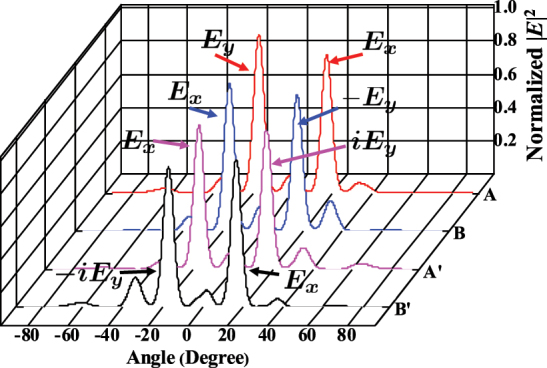
Simulation results of the far-filed transmission spectra excited by a normally incident light with ***E***_*x*_ + ***E***_*y*_ (i.e., -polarized) on the pixels A, B, $A^{\prime }$, and $B^{\prime }$. The output light is divided into −1 order and +1 order in the far-field. For the pixels A, B, $A^{\prime }$, and $B^{\prime }$, the output light beams in +1(−1) order are ***E***_*y*_(***E***_*x*_), ***E***_*x*_(−***E***_*y*_), *i* ***E***_*y*_(***E***_*x*_), and ***E***_*x*_(−*i* ***E***_*y*_), respectively.

Calculation for the far-field distribution of each pixel with supercells A, B, $A^{\prime }$, and $B^{\prime }$ operating in periodic boundary conditions is illustrated in Figure 4. As expected, the pixel with supercell A(B) splits the light with ***E***_*y*_(***E***_*x*_) and ***E***_*x*_(−***E***_*y*_) in the left and the right paths, respectively, while the pixel with supercell $A^{\prime }$($B^{\prime }$) splits the light with −*i* ***E***_*y*_(***E***_*x*_) and ***E***_*x*_(*i* ***E***_*y*_) in the left and the right paths, respectively. Illuminated by the light with the electric field given by ***E***_*x*_ + ***E***_*y*_, the combination of pixels with the supercell A and B can be used to achieve a ***E***_*x*_ + ***E***_*y*_ response for the left path and a ***E***_*x*_ − ***E***_*y*_ response for the right path. The combination of pixels with supercell $A^{\prime }$ and $B^{\prime }$ can be used to achieve a ***E***_*x*_ − *i* ***E***_*y*_ response for the left path and a ***E***_*x*_ + *i* ***E***_*y*_ response for the right path. This behavior persist for any incident polarization as needed for the BSM.

## 3 Metasurface-based Bell states measurement

**Figure 5:**
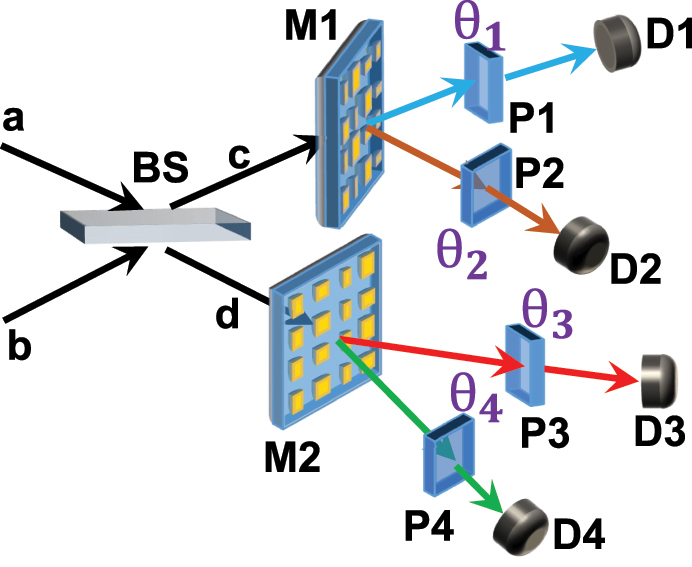
Sketch of metasurface-enhanced Innsbruck scheme for BSM relying on fixed metasurfaces. The entangled photon pair injects the system by the two separated paths *a* and *b*. On each path, the metasurface splits the photons into different polarization state. The polarizers *P*1 and *P*2 are placed before the detectors to perform the orientation dependent projection.

As we have mentioned before, the Innsbruck BSM scheme containing a 50/50 BS and the two PBS allows to distinguish only two out of the all four Bell states. In our scheme, the two PBS are replaced by the two binary-pixel metasurface *M*1 and *M*2 in paths *c* and *d* as shown in Figure 5. These two photons coming from the input modes *a* and *b* can be described by the four photon annihilation operators *a*_*x*_, *a*_*y*_, *b*_*x*_, *b*_*y*_, where *a* and *b* are the path modes, and *x* and *y* denote the polarization mode. The four Bell states originated from these two photons can be written as

(9) $$\begin{array}{l} \begin{array}{cc} |\Psi_{1}〉 & =\frac{1}{\sqrt{2}}\left({\hat{a}}_{x}^{†}{\hat{b}}_{y}^{†}-{\hat{a}}_{y}^{†}{\hat{b}}_{x}^{†}\right)|0〉,\\ |\Psi_{2}〉 & =\frac{1}{\sqrt{2}}\left({\hat{a}}_{x}^{†}{\hat{b}}_{y}^{†}+{\hat{a}}_{y}^{†}{\hat{b}}_{x}^{†}\right)|0〉,\\ |\Psi_{3}〉 & =\frac{1}{\sqrt{2}}\left({\hat{a}}_{x}^{†}{\hat{b}}_{x}^{†}-{\hat{a}}_{y}^{†}{\hat{b}}_{y}^{†}\right)|0〉,\\ |\Psi_{4}〉 & =\frac{1}{\sqrt{2}}\left({\hat{a}}_{x}^{†}{\hat{b}}_{x}^{†}+{\hat{a}}_{y}^{†}{\hat{b}}_{y}^{†}\right)|0〉, \end{array} \end{array}$$

where |0⟩ is the vacuum state. For simplicity, it is assumed that the BS imparts an equal phase shifts to each polarization, so that the BS transformations connecting the input photons with the two output photons are

(10) $$\begin{array}{c} \hat{c}=\left(\hat{a}+\hat{b}\right)/\sqrt{2},\;\hat{d}=\left(\hat{a}-\hat{b}\right)/\sqrt{2}, \end{array}$$

where the polarization indices are omitted. In *c* path, the metasurface *M*1 is used to split the *c* mode photon into the detectors *D*1 and the *D*2. Between the metasurface *M*1 and the detectors, the polarizers are placed in the middle to project the photon onto the polarization state cos *θ* ***e***_*x*_ + sin *θ* ***e***_*y*_ controlled by the rotation angle of polarizers *θ*_1_ and *θ*_2_. The angles that enter the amplitude coefficients *α*_*i*_ (*i* = 1, 2, 3, 4) in Eqs. (7) and (8) are set to be *π*/4. If the imperfection is considered, the amplitude coefficients are changed as shown in the supplementary material. The detected fields at *D*1 and *D*2 satisfy the following relation with the amplitudes

(11) $$\begin{array}{l} \begin{array}{cc} {\hat{D}}_{1} & =\left({\hat{c}}_{x}\cos\theta_{1}+{\hat{c}}_{y}\sin\theta_{1}\right)/\sqrt{2},\\ {\hat{D}}_{2} & =\left({\hat{c}}_{x}\sin\theta_{2}-{\hat{c}}_{y}\cos\theta_{2}\right)/\sqrt{2}. \end{array} \end{array}$$

In another path after the BS, the photon passes though the metasurface *M*2. The photon state is then projected by the polarizers governed by the angles *θ*_3_ and *θ*_4_ before the detection at the detectors *D*3 and *D*4. The input–output relations between the detected fields *D*3, *D*4 and the incoming amplitudes *d*_*x*,*y*_ read

(12) $$\begin{array}{l} \begin{array}{cc} {\hat{D}}_{3} & =\left({\hat{d}}_{x}\cos\theta_{3}+i{\hat{d}}_{y}\sin\theta_{3}\right)/\sqrt{2},\\ {\hat{D}}_{4} & =\left({\hat{d}}_{x}\cos\theta_{4}-i{\hat{d}}_{x}\sin\theta_{4}\right)/\sqrt{2}. \end{array} \end{array}$$

We now calculate the two-photon amplitudes corresponding to the two-photon coincidence counting signals. Firstly, the two-photon coincidence counting signals from both modes *c* and *d* only give the antisymmetric Bell state |Ψ_1_⟩. They are

(13) $$\begin{array}{l} \begin{array}{cc} 〈{\hat{D}}_{13}| & =\left(\sin\theta_{1}\cos\theta_{3}-i\cos\theta_{1}\sin\theta_{3}\right)〈\Psi_{1}|/\left(2\sqrt{2}\right),\\ 〈{\hat{D}}_{14}| & =\left(\sin\theta_{1}\cos\theta_{4}+i\cos\theta_{1}\sin\theta_{4}\right)〈\Psi_{1}|/\left(2\sqrt{2}\right),\\ 〈{\hat{D}}_{23}| & =-\left(\sin\theta_{2}\cos\theta_{3}+i\cos\theta_{2}\sin\theta_{3}\right)〈\Psi_{1}|/\left(2\sqrt{2}\right),\\ 〈{\hat{D}}_{24}| & =-\left(\sin\theta_{2}\cos\theta_{4}-i\cos\theta_{2}\sin\theta_{4}\right)〈\Psi_{1}|/\left(2\sqrt{2}\right). \end{array} \end{array}$$

Secondly, the two-photon coincidence counting signals in each path of *c* and *d* give two different linear combination of |Ψ_2_⟩, |Ψ_3_⟩, and |Ψ_4_⟩. The exact forms are

(14) $$\begin{array}{l} \begin{array}{cc} & 〈{\hat{D}}_{12}|=\left(\cos\theta_{12}^{-}〈\Psi_{2}|+\sin\theta_{12}^{-}〈\Psi_{3}|+\sin\theta_{12}^{+}〈\Psi_{4}|\right)/\left(2\sqrt{2}\right),\\ & 〈{\hat{D}}_{34}|=-\left(i\cos\theta_{34}^{-}〈\Psi_{2}|+\sin\theta_{34}^{+}〈\Psi_{3}|+\sin\theta_{34}^{-}〈\Psi_{4}|\right)/\left(2\sqrt{2}\right). \end{array} \end{array}$$

Hereafter, $\theta_{ij}^{+}=\theta_{i}+\theta_{j},\theta_{ij}^{-}=\theta_{i}-\theta_{j}$. The two-photon amplitudes $〈{\hat{D}}_{12}|$ and $〈{\hat{D}}_{34}|$ indicate that the three Bell states dependent on the three different angles. For $〈{\hat{D}}_{12}|$, since $\cos\theta_{12}^{-}\neq \sin\theta_{12}^{-}=0$, it makes Bell state ⟨Ψ_4_| indistinguishable in this measurement. For $〈{\hat{D}}_{34}|$, $\cos\theta_{34}^{-}\neq \sin\theta_{34}^{-}=0$ results in the indiscernibility of Bell state ⟨Ψ_3_|. Thus, |Ψ_2_⟩, |Ψ_3_⟩, and |Ψ_4_⟩ can be individually identified by combining the two measurements together.

Furthermore, if the two-photon coincidence counting signals detected in the same detector, they are given by the linear combination of |Ψ_2,3,4_⟩, which cannot be used to identify any Bell state separately. The corresponding projections are

(15) $$\begin{array}{l} \begin{array}{cc} 〈{\hat{D}}_{11}| & =\left(〈\Psi_{4}|+\cos2\theta_{1}〈\Psi_{3}|+\sin2\theta_{1}〈\Psi_{2}|\right)/\left(2\sqrt{2}\right),\\ 〈{\hat{D}}_{22}| & =\left(〈\Psi_{4}|+\cos2\theta_{2}〈\Psi_{3}|-\sin2\theta_{21}〈\Psi_{2}|\right)/\left(2\sqrt{2}\right),\\ 〈{\hat{D}}_{33}| & =-\left(〈\Psi_{3}|+\cos2\theta_{3}〈\Psi_{4}|+i\sin2\theta_{3}〈\Psi_{2}|\right)/\left(2\sqrt{2}\right),\\ 〈{\hat{D}}_{44}| & =\left(-〈\Psi_{3}|-\cos2\theta_{3}〈\Psi_{4}|+i\sin2\theta_{3}〈\Psi_{2}|\right)/\left(2\sqrt{2}\right). \end{array} \end{array}$$

If the input two-photon state in an arbitrary linear superposition of all four Bell states *α*|Ψ_1_⟩ + *β*|Ψ_2_⟩ + *γ*|Ψ_3_⟩ + *δ*|Ψ_4_⟩ is measured by our BSM setup, where ${\left|\alpha \right|}^{2}+{\left|\beta \right|}^{2}+{\left|\gamma \right|}^{2}+{\left|\delta \right|}^{2}=1$. By using the projection in Eqs. (13)–(15), one can obtain the coincidence counting measurements $G_{ij}^{\left(2\right)}\left(\Psi_{k}\right)=|\left\langle {\hat{D}}_{ij}|\Psi_{k}\right\rangle {|}^{2}$ where *i*, *j*, *k* = 1, 2, 3, 4. For instance, antisymmetric Bell state Ψ_1_ can be measured in the following outcomes with the given probabilities

(16) $$\begin{array}{c} G_{jk}^{\left(2\right)}\left(\Psi_{1}\right)={\left|\alpha \right|}^{2}\left(1-\sin2\theta_{j}\cos2\theta_{k}\right)/16 \end{array}$$

**Figure 6:**
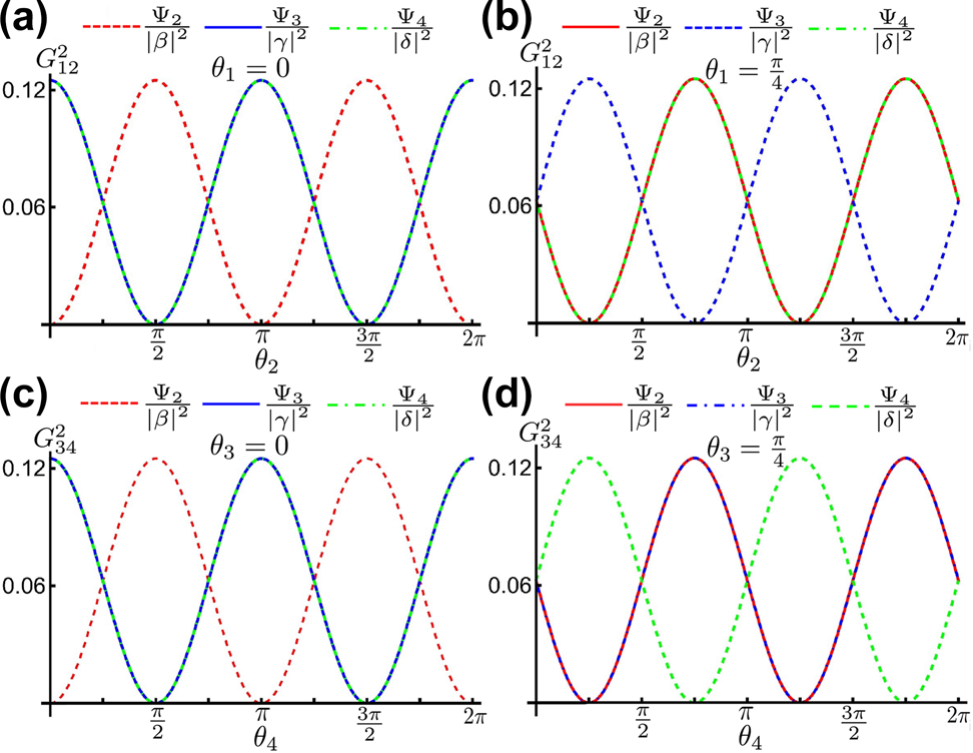
Coincidence counting outcomes for measurement of asymmetric Bell states |Ψ_2_⟩, symmetric Bell states |Ψ_3_⟩, |Ψ_4_⟩. (a, b) For coincidence counting measurements $G_{12}^{\left(2\right)}$, the detection probability of Ψ_2_ shows anticorrelation with Ψ_3_ and Ψ_4_ as *θ*_1_ = 0, while the detection probability of Ψ_3_ shows anticorrelation with Ψ_2_ and Ψ_3_ as *θ*_1_ = *π*/4. (c, d) For coincidence counting measurements $G_{34}^{\left(2\right)}$, the probability of Ψ_2_ is anticorrelated with that of Ψ_3_ and Ψ_2_ as *θ*_4_ = 0, while the detection probability of Ψ_4_ is anticorrelated with that of Ψ_2_ and Ψ_3_ as *θ*_3_ = *π*/4.

The coincidence counting measurements in both detectors *D*1 and *D*2 reveals that |Ψ_2,3_⟩ can be distinguished in different measurements by setting the rotating angles as $\cos\theta_{12}^{-}=0$ or $\sin\theta_{12}^{-}=0$. The corresponding coincidence counting measurements are $G_{12}^{\left(2\right)}\left(\Psi_{2}\right)={\left|\beta \right|}^{2}\sin^{2}\theta_{12}^{-}/8$ and $G_{12}^{\left(2\right)}\left(\Psi_{3}\right)={\left|\gamma \right|}^{2}\cos^{2}\theta_{12}^{-}/8$. As shown in Figure 6(a) and (b), the $G_{12}^{\left(2\right)}\left(\Psi_{2}\right)$ is anticorrelated with $G_{12}^{\left(2\right)}\left(\Psi_{3}\right)$ and $G_{12}^{\left(2\right)}\left(\Psi_{4}\right)$ as *θ*_1_ = 0. Then, Ψ_2_ can be discriminated by choosing the rotation angle *θ*_2_ = (*n* + 1)*π*/2 with *n* = 0, ±1, ±2, ⋯. While $\theta_{1}=\frac{\pi }{4}$, $G_{12}^{\left(2\right)}\left(\Psi_{3}\right)$ is anticorrelated with $G_{12}^{\left(2\right)}\left(\Psi_{2}\right)$ and $G_{12}^{\left(2\right)}\left(\Psi_{4}\right)$ which makes Ψ_3_ identifiable as *θ*_2_ = (2*n* + 1)*π*/4 with *n* = 0, ±1, ±2, ⋯. The coincidence counting in detectors *D*3 and *D*4 shows the discrimination of another pair of Bell states |Ψ_2_⟩ and |Ψ_4_⟩ with the coincidence counting measurements $G_{34}^{\left(2\right)}\left(\Psi_{2}\right)={\left|\beta \right|}^{2}\sin^{2}\theta_{34}^{-}/8$ and $G_{34}^{\left(2\right)}\left(\Psi_{4}\right)={\left|\delta \right|}^{2}\cos^{2}\theta_{34}^{-}/8$. As shown in Figure 6(c) and (d), $G_{34}^{\left(2\right)}\left(\Psi_{4}\right)$ is anticorrelated with $G_{34}^{\left(2\right)}\left(\Psi_{2}\right)$ and $G_{34}^{\left(2\right)}\left(\Psi_{3}\right)$ resulting in the discrimination of Ψ_4_, while |Ψ_2_⟩ is isolated as *θ*_3_ = 0. These angle-controlled state anticorrelations demonstrate that this metasurface-enhanced Innsbruck scheme can be successfully applied to distinguish each Bell state from an arbitrary input with the linear superposition of four Bell states. According to the above discussion, three out of four Bell states are distinguished independently in a single measurement as shown in the following examples. Bell states *ψ*_1_, *ψ*_2_, and *ψ*_3_ can be simultaneously measured in *G*_13_, *G*_14_, *G*_23_, *G*_24_, *G*_34_, and *G*_12_ respectively, when the angle of polarizers are set as *θ*_1_ = *π*/4, *θ*_2_ = *π*/4, *θ*_3_ = 0, and *θ*_4_ = *π*/2. Bell states *ψ*_1_, *ψ*_2_, and *ψ*_4_ can be simultaneously measured in *G*_13_, *G*_14_, *G*_23_, *G*_24_, *G*_12_, and *G*_34_, respectively, when the angle of polarizers are set as *θ*_1_ = 0, *θ*_2_ = *π*/2, *θ*_3_ = *π*/4, and *θ*_4_ = *π*/2. Considering the detection probability of antisymmetric Bell state Ψ_1_ shown in Eq. (16), all of four Bell states can reach the same maximum detection probability, which makes the metasurface-enhanced Innsbruck scheme more intuitive to reconstruct the superposition of four Bell states. However, the fabrication imperfection of metasurface can affect the outcome of Bell state measurement as discussed in the supplementary material.

## 4 Summary

We theoretically propose a metasurface-enhanced Innsbruck scheme for complete BSM. The key distinctive feature of this approach is to simplify the level of complexity and to reduce the number of excessive bulk optical elements generally used in quantum optics setup. The polarization engineering capabilities of metasurfaces are exploited to directly perform a high efficiency manipulation of polarization state of light in two output paths, aiming at a tunable superposition of symmetric Bell states in the coincidence counting measurements. Instead of directly detecting polarization state of the photon using the intensity measurement of a single state, as it is usually performed in a normal projection measurement, the polarization superposition provided by the metasurface achieves a direct detection of the polarization Bell state. In a way, we propose to directly probe the two-photon state entanglement relying on a direct coincidence counting measurement technique, which appears to be only accessible in the specifically designed dual-polarization mode metasurface. Our approach thereby achieves a complete BSM by adjusting only the angle of polarizers used for the quantum state projection, which not only actualizes the discrimination of all four Bell states but also enables the exact same setup to perform simultaneous collection, without adding or suppressing any optical element. In addition, the complete counting statistics of all four Bell states collected in a single experiment allows to properly project the incoming state on a superposition of the Bell states. One could thus explore multiple quantum interference effects due to multiple detection pathways with different phases determined by the rotating angles *θ*_*i*_ (*i* = 1, 2, 3, 4). Our results have potential for implementation in quantum dense coding, entanglement swapping, quantum teleportation protocols, quantum secure direct communications, and other quantum technologies requiring precise and robust setups with minimally adjustable optical elements. Our work shows that the metasurfaces can be designed to act on a photon wavepacket at the subwavelength level while conventional PBS/BS are acting on the entire photon wavepacket, a key feature that extends the utilization of metasurfaces in the realm of quantum state measurements. Achieving the subwavelength mixed photon manipulation with the multiplexed metasurfaces offers new appealing solutions for precise and on-chip implementation of quantum optics measurements [48], [49], [50], [51], [52], [53].

## Supplementary Material

Supplementary Material Details
