# Supplementary material for: Metasurface for complete measurement of polarization Bell state
Source: Nanophotonics. 2022 Nov 21;12(3):569–77. doi: 10.1515/nanoph-2022-0593 (PMC11501330; doi:10.1515/nanoph-2022-0593)
Supplement: Supplementary file 1 — Supplementary Material Details [file j_nanoph-2022-0593_suppl_001.pdf]

Supplementary material - Metasurface for complete measurement of polarization Bell state

Zhanjie Gao,[1] Zengping Su,[2] Qinghua Song,[2,*] Patrice Genevet,[3,†] and Konstantin E. Dorfman[1,4,5,‡]

[1] *State Key Laboratory of Precision Spectroscopy, East China Normal University, Shanghai 200062, China*

[2] *Tsinghua Shenzhen International Graduate School, Tsinghua University, Shenzhen, 518055, China*

[3] *Université Côte d'Azur, CNRS, CRHEA, rue Bernard Gregory, 06560 Valbonne, France*

[4] *Collaborative Innovation Center of Extreme Optics, Shanxi University, Taiyuan, Shanxi 030006, China*

[5] *Himalayan Institute for Advanced Study, Unit of Gopinath Seva Foundation, MIG 38, Avas Vikas, Rishikesh, Uttarakhand 249201, India*

I. THE MODIFICATION DUE TO THE FABRICATION DEFECT

In the real fabrication setup, there always exist various fabrication imperfections. Thus, the effects of fabrication imperfection have to be included. In this supplementary material, we discuss the detection error in the Bell state measurement resulting from the loss and changes in the transmission efficiency in different scattering channels.

As shown in the main text, considering the photon number conservation and normalization, the relation between the photons in the input and the ± 1th order channels for metasurface $M1$ can be written as

$$\begin{aligned} \hat{a}_{1,x} &= \cos\alpha_1 \hat{a}_x, \quad \hat{a}_{1,y} = -\cos\alpha_2 \hat{a}_y, \\ \hat{a}_{-1,x} &= \sin\alpha_1 \hat{a}_x, \quad \hat{a}_{-1,y} = \sin\alpha_2 \hat{a}_y. \end{aligned} \tag{1}$$

Here, the parameters α_1 and α_2 denote the transmission efficiency in different scattering channels for the x and y polarizations. In the present Bell state measurement scheme, the metasurface is designed that the energy is divided equally between the 1 and -1 scattering orders with $\alpha_1 = \alpha_2 = \pi/4$.

In the presence of the losses or the existence of other undesired scattering processes, Eq. (1) has to be modified, which yields

$$\begin{aligned} \hat{a}_{1,x} &= A_1 \cos\alpha_1 \hat{a}_x + \delta 1 \hat{a}_y, \quad \hat{a}_{1,y} = -A_2 \cos\alpha_2 \hat{a}_y + \delta 2 \hat{a}_x, \\ \hat{a}_{-1,x} &= A_1 \sin\alpha_1 \hat{a}_x + \delta 3 \hat{a}_y, \quad \hat{a}_{-1,y} = A_2 \sin\alpha_2 \hat{a}_y + \delta 4 \hat{a}_x, \end{aligned} \tag{2}$$

where coefficients satisfy $|A_1|^2 + |\delta 2|^2 + |\delta 4|^2 \leq 1$, $|A_2|^2 + |\delta 1|^2 + |\delta 3|^2 \leq 1$ where the inequality indicates the loss of energy in the ± 1 scattering orders. It should be mentioned that the metasurfaces $M1$ and $M2$ depend on the propagation phase which does not need to change the orientation of antennas in the fabrication process. Thus, one can assign the long and short sides of the nano-antennas along x and y directions. The fabrication process can fix precisely the orientation of the nano antennas. The main imperfection rather comes from the error in the fabrication of a perfect rectangular shape. Since the orientation of each antenna is controlled with high precision, there is no cross polarization conversion, which makes $\delta_{1,2,3,4} = 0$. In the following, we therefore omit all the cross polarization conversion terms.

For the metasurface $M2$, the input-output relation is modified into

$$\begin{aligned} \hat{a}_{1,x} &= A_3 \cos\alpha_3 \hat{a}_x, \quad \hat{a}_{1,y} = -iA_4 \cos\alpha_4 \hat{a}_y, \\ \hat{a}_{-1,x} &= A_3 \sin\alpha_3 \hat{a}_x, \quad \hat{a}_{-1,y} = iA_4 \sin\alpha_4 \hat{a}_y. \end{aligned} \tag{3}$$

where coefficients $|A_{3,4}|^2 \leq 1$.

* song.qinghua@sz.tsinghua.edu.cn
† Patrice.Genevet@crhea.cnrs.fr
‡ dorfmank@lps.ecnu.edu.cn

II. THE EFFECT ORIGINATING FROM FABRICATION IMPERFECTION

Considering the fabrication imperfections, the two photon coincidence counting signals from the modes c and d reads

$$
\begin{aligned}
\langle \hat{D}_{13}| &= (A_2A_3\cos\alpha_2\cos\alpha_3\sin\theta_1\cos\theta_3 - iA_1A_4\cos\alpha_1\cos\alpha_4\cos\theta_1\sin\theta_3)\langle\Psi_1|/\sqrt{2},\\
\langle \hat{D}_{14}| &= (A_2A_3\cos\alpha_2\sin\alpha_3\sin\theta_1\cos\theta_4 + iA_1A_4\cos\alpha_1\sin\alpha_4\cos\theta_1\sin\theta_4)\langle\Psi_1|/\sqrt{2},\\
\langle \hat{D}_{23}| &= -(A_2A_3\sin\alpha_2\cos\alpha_3\sin\theta_2\cos\theta_3 + iA_1A_4\sin\alpha_1\cos\alpha_4\cos\theta_2\sin\theta_3)\langle\Psi_1|/\sqrt{2},\\
\langle \hat{D}_{24}| &= -(A_2A_3\sin\alpha_2\sin\alpha_3\sin\theta_2\cos\theta_4 - iA_1A_4\sin\alpha_1\sin\alpha_4\cos\theta_2\sin\theta_4)\langle\Psi_1|/\sqrt{2}.
\end{aligned}
\tag{4}
$$

While Eq. (4) still gives an unambiguous measurement of state $|\Psi_1\rangle$, the fabrication imperfection reduces the detection probability of $|\Psi_1\rangle$, when $|A_{1,2,3,4}|$ is smaller than 1.

We similarly obtain for the $\langle\hat{D}_{12}|$ and $\langle\hat{D}_{34}|$

$$
\begin{aligned}
\langle \hat{D}_{12}| =&[A_1A_2(\sin\alpha_1\cos\alpha_2\sin\theta_1\cos\theta_2 - \cos\alpha_1\sin\alpha_2\cos\theta_1\sin\theta_2)\langle\Psi_2|\\
&+ (A_1^2\cos\alpha_1\sin\alpha_1\cos\theta_1\cos\theta_2 + A_2^2\cos\alpha_2\sin\alpha_2\sin\theta_1\sin\theta_2)\langle\Psi_3|\\
&+ (A_1^2\cos\alpha_1\sin\alpha_1\cos\theta_1\cos\theta_2 - A_2^2\cos\alpha_2\sin\alpha_2\sin\theta_1\sin\theta_2)\langle\Psi_4|]/\sqrt{2},\\
\langle \hat{D}_{34}| =&[-iA_3A_4(\sin\alpha_3\cos\alpha_4\sin\theta_3\cos\theta_4 - \cos\alpha_3\sin\alpha_4\cos\theta_3\sin\theta_4)\langle\Psi_2|\\
&+ (-A_3^2\cos\alpha_3\sin\alpha_3\cos\theta_3\cos\theta_4 + A_4^2\cos\alpha_4\sin\alpha_4\sin\theta_3\sin\theta_4)\langle\Psi_3|\\
&- (A_3^2\cos\alpha_3\sin\alpha_3\cos\theta_3\cos\theta_4 + A_4^2\cos\alpha_4\sin\alpha_4\sin\theta_3\sin\theta_4)\langle\Psi_4|]/\sqrt{2}.
\end{aligned}
\tag{5}
$$

Equation (5) yields the linear superposition of $|\Psi_2\rangle$, $|\Psi_3\rangle$, and $|\Psi_4\rangle$ which is different from the ideal metasurface case. In the main text, it is shown that the Bell states Ψ_2 and $\Psi_{3,4}$ are anti-correlated with the rotation angle parameters $\theta_{1,3} = 0$. When θ_1 changes from 0 to $\pi/4$ in $G^{(2)}_{12}$, $|\Psi_2\rangle$, and $|\Psi_4\rangle$ are correlated, while $|\Psi_3\rangle$ is anti-correlated with $|\Psi_{3,4}\rangle$. When θ_3 changes from 0 to $\pi/4$ in $G^{(2)}_{34}$, $|\Psi_2\rangle$, and $|\Psi_3\rangle$ are correlated, while $|\Psi_4\rangle$ is anti-correlated with $|\Psi_{2,3}\rangle$. Thus, we can distinguish three out of four Bell states in one measurement, and distinguish the remaining one by rotating the angle of polarizer in additional measurement. In order to analyze the impact of fabrication imperfection, we can control the parameters θ_1 or θ_3 changes from 0 to $\pi/4$ to see changes of the detection of Ψ_2, Ψ_3 and Ψ_4.

When $\theta_1 = 0$ and $\theta_3 = 0$, the two photon coincidence counting signals yield

$$
\begin{aligned}
\langle \hat{D}_{12}| =&(-A_1A_2\cos\alpha_1\sin\alpha_2\sin\theta_2\langle\Psi_2| + A_1^2\cos\alpha_1\sin\alpha_1\cos\theta_2\langle\Psi_3| + A_1^2\cos\alpha_1\sin\alpha_1\cos\theta_2\langle\Psi_4|)/\sqrt{2},\\
\langle \hat{D}_{34}| =&(iA_3A_4\cos\alpha_3\sin\alpha_4\sin\theta_4\langle\Psi_2| - A_3^2\cos\alpha_3\sin\alpha_3\cos\theta_4\langle\Psi_3| - A_3^2\cos\alpha_3\sin\alpha_3\cos\theta_4\langle\Psi_4|)/\sqrt{2}.
\end{aligned}
\tag{6}
$$

It is clear that Ψ_3 and Ψ_4 retain their correlation relation and Ψ_2 and $\Psi_{3,4}$ shows anti-correlation relation if one tunes the $\theta_{2,4}$. Thus,the imperfection only diminishes the detection probability, but does not affect the identification of Bell state Ψ_2.

The two photon coincidence counting signals with $\theta_1 = \pi/4$ and $\theta_3 = \pi/4$ become

$$
\begin{aligned}
\langle \hat{D}_{12}| =&[A_1A_2(\sin\alpha_1\cos\alpha_2\cos\theta_2 - \cos\alpha_1\sin\alpha_2\sin\theta_2)\langle\Psi_2|\\
&+ (A_1^2\cos\alpha_1\sin\alpha_1\cos\theta_2 + A_2^2\cos\alpha_2\sin\alpha_2\sin\theta_2)\langle\Psi_3|\\
&+ (A_1^2\cos\alpha_1\sin\alpha_1\cos\theta_2 - A_2^2\cos\alpha_2\sin\alpha_2\sin\theta_2)\langle\Psi_4|]/2,\\
\langle \hat{D}_{34}| =&[-iA_3A_4(\sin\alpha_3\cos\alpha_4\cos\theta_4 - \cos\alpha_3\sin\alpha_4\sin\theta_4)\langle\Psi_2|\\
&+ (-A_3^2\cos\alpha_3\sin\alpha_3\cos\theta_4 + A_4^2\cos\alpha_4\sin\alpha_4\sin\theta_4)\langle\Psi_3|\\
&- (A_3^2\cos\alpha_3\sin\alpha_3\cos\theta_4 + A_4^2\cos\alpha_4\sin\alpha_4\sin\theta_4)\langle\Psi_4|]/2.
\end{aligned}
\tag{7}
$$

It is clear that the anti-correlation relation between the Bell states Ψ_3 and Ψ_4 is not retained, and Ψ_2 is no longer correlated with Ψ_3 (Ψ_4) in two photon coincidence counting signals $G^{(2)}_{12}$ ($G^{(2)}_{34}$), which makes the determination of Bell states Ψ_3 and Ψ_4 deviate from the idea case. Because the two photon coincidence counting $G^{(2)}_{12}$ and $G^{(2)}_{34}$ have the same dependence on the control parameters, only the two photon coincidence counting $G^{(2)}_{12}$ is discussed as an example blow.

Since the Bell state Ψ_3 is distinct as $\theta_1 = \theta_2 = \pi/4$ for the ideal metasurface setup in two photon coincidence counting $G^{(2)}_{12}$. In order to analyze the impact on the Bell states detection accuracy due to the fabrication imperfection, the

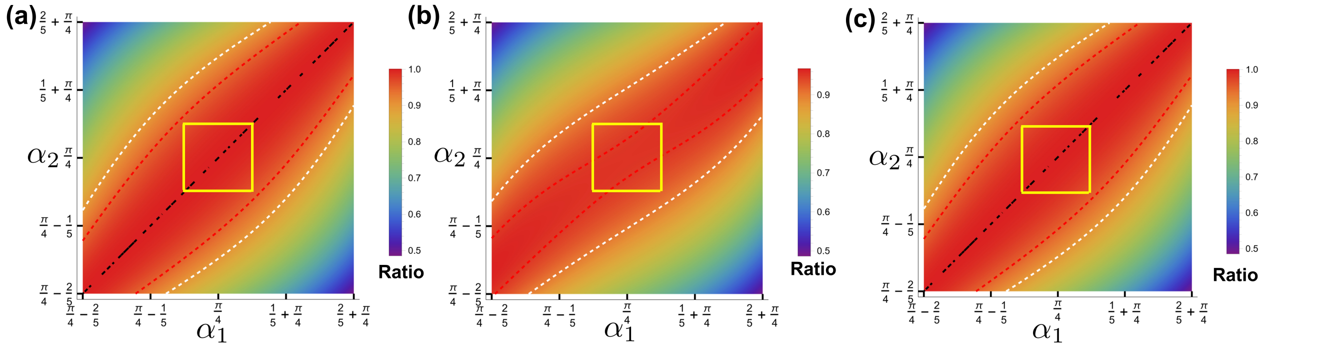

FIG. 1. The contribution of Bell state $|\Psi_3\rangle$ in the coincidence counting outcomes $G_{12}^{(2)}$ as a function of $\alpha_{1,2}$ parameters at fixed rotation angle $\theta_1 = \theta_2 = \pi/4$. The black, read and white dashed line denotes the contribution of 100%, 95% and 90% respectively. The yellow box denotes $\pi/4 - 1/10 \leq \alpha_{1,2} \leq \pi/4 + 1/10$. For the coincidence counting measurements $G_{12}^{(2)}$, the contribution of Bell states $|\Psi_3\rangle$ is changed as the $\pi/4 - 2/5 \leq \alpha_{1,2} \leq \pi/4 + 2/5$. The remaining parameters are: (a) $A_1 = A_2 = 1$, (b) $A_1 = 1$, $A_2 = 4/5$, (c) $A_1 = 4/5$, $A_2 = 4/5$.

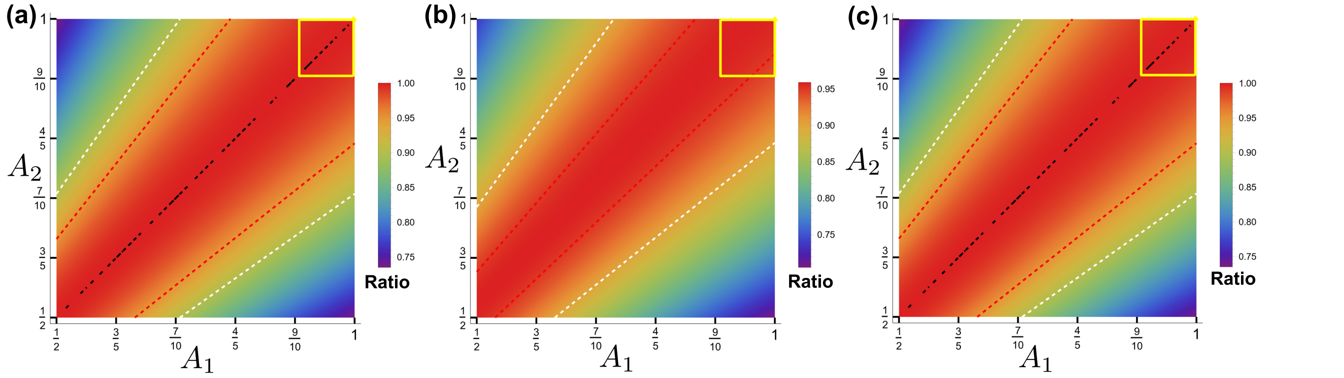

FIG. 2. The contribution of Bell state $|\Psi_3\rangle$ in the coincidence counting outcomes $G_{12}^{(2)}$ as a function of $A_{1,2}$ parameters at fixed rotation angle $\theta_1 = \theta_2 = \pi/4$. The black, read and white dashed line denotes the contribution of 100%, 95% and 90% respectively. The yellow box denotes $0.9 \leq A_{1,2} \leq 1$. For the coincidence counting measurements $G_{12}^{(2)}$, the contribution of Bell states $|\Psi_3\rangle$ is changed as the $1/2 \leq A_{1,2} \leq 1$. The remaining parameters are: (a) $\alpha_1 = \alpha_2 = \pi/4$, (b) $\alpha_1 = \pi/4$, $\alpha_2 = \pi/4 - 1/10$, (c) $\alpha_1 = \pi/4 - 1/10$, $\alpha_2 = \pi/4 - 1/10$.

contribution of target Bell state Ψ_3 in the coincidence counting $G_{12}^{(2)}$ is calculated at $\theta_1 = \theta_2 = \pi/4$. In Figs. 1 and 2, the contribution of target Bell state Ψ_3 is highly sensitive to parameters $\alpha_{1,2}$ and $A_{1,2}$. As shown in Fig. 1(a), the contribution of Ψ_3 changes from 100% to 48.54% when the offset between $\alpha_{1,2}$ and $\pi/4$ varies from 0 to 2/5. If we consider an offset smaller than 1/10 where the energy distribution difference between the two channels is smaller than 1%, the contribution of Ψ_3 is larger than 95% as shown in the yellow box of Fig. 1(a). If $\alpha_1 = \alpha_2$, the contribution of Ψ_3 is fixed at 100%. As shown in Fig. 1(b), if the loss in x and y polarization components is different, the contribution of Ψ_3 decays faster than the lossless case, and can not reach 100%. If the loss in x and y polarization components is the same ($A_1 = A_2 \leq 1$), the changes of the contribution of Ψ_3 is the same as lossless case. Figure 2 shows the changes of the contribution of Ψ_3 when the loss results in A_1 and A_2 varying from 1 to 1/2. It is clear that the decrease of A_1 or A_2 decreases the contribution of Ψ_3. However, if the loss is the same for x and y polarization, the contribution of Ψ_3 remains at 100% shown in Fig. 2(a). If we consider a small offset smaller than 1/10 where the loss in both channels is smaller than 1%, the contribution of Ψ_3 is larger than 95% as shown in the yellow box of Fig. 2(a). Figure 2(b) shows that the different energy distribution between the x and y polarization in different channels makes the contribution of Ψ_3 below 100%. However, as shown in Fig. 2(c), when the energy distribution in different channels is the same ($\alpha_1 = \alpha_2 \leq \pi/4$), the changes of the contribution of Ψ_3 is the same as them 50/50 energy distribution case illustrated in the Fig. 2(a).

In summary, our Bell state measurement scheme is highly dependent on the loss and energy distribution between different channels coming from the fabrication imperfection of the metasurface. However, if the error is smaller than 1%, the Bell states measurement can have high accuracy where the contribution of target Bell states is larger than 95%. In the meantime, if the loss and the other imperfection can not be perfectly match the ideal condition, a careful fabrication results in transmission efficiency of pixels have relation $A_1 = A_2$, $A_3 = A_4$, $\alpha_1 = \alpha_2$ and $\alpha_3 = \alpha_4$, which can make the contribution of target Bell states reach 100%.
